# Surgical sequence in anterior column realignment with posterior osteotomy is important for degree of adult spinal deformity correction: advantages and indications for posterior to anterior sequence

**DOI:** 10.1186/s12891-022-05915-4

**Published:** 2022-11-22

**Authors:** Sung-Min Kim, Yong-Chan Kim, Ki-Tack Kim, Kee-Yong Ha, Qiang Luo, Xiongjie Li, JunBum Park

**Affiliations:** 1 Department of Orthopaedic Surgery, College of Medicine, Kyung Hee University Hospital at Gangdong, Kyung Hee University, 892 Dongnam-ro, Gangdong-gu, 05278 Seoul, Korea; 2 Department of Orthopaedic Surgery, Graduate School of Medicine, Kyung Hee University, Kyungheedae-ro, Dongdaemun-gu, Seoul, Korea

**Keywords:** Adult spinal deformity, Posterior osteotomy, Anterior column realignment, Surgical sequence

## Abstract

**Background:**

We hypothesized that posterior osteotomy prior to ACR (Anterior column realignment) through P-A-P surgical sequence would permit a greater correction for deformity corrective surgery than the traditional A-P sequence without posterior osteotomy. This study aimed to determine the impact of the P-A-P sequence on the restoration of lumbar lordosis (LL) compared to the A-P sequence in deformity corrective surgery for adult spinal deformity (ASD) patients and to identify the characteristics of patients who require this sequence.

**Methods:**

Between 2017 and 2019, 260 ASD patients who had undergone combined corrective surgery were reviewed retrospectively. This study included 178 patients who underwent posterior osteotomy before the ACR (P-A group) and 82 patients who underwent the A-P sequence (A-P group). Sagittal spinopelvic parameters were determined from pre- and postoperative whole-spine radiographs and compared between the groups. To find better indications for the P-A-P sequence, we conducted additional analysis on postoperative outcomes of patients in the A-P group.

**Results:**

The P-A group showed a significantly higher change in LL (53.7° vs. 44.3°, *p* < 0.001), C7 sagittal vertical axis (C7 SVA: 197.4 mm vs. 146.1 mm, *p* = 0.021), segmental lordosis (SL) L2/3 (16.2° vs. 14.4°, *p* = 0.043), SL L3/4 (16.2° vs. 13.8°, *p* = 0.004), and SL L4/5 (15.1° vs. 11.3°, *p* = 0.001) compared to the A-P group. At the final follow-up, pelvic incidence (PI) minus LL mismatch (PI − LL mismatch) was significantly higher in the A-P group (13.4° vs. 2.9°, *p* < 0.001). Stepwise logistic regression analysis showed that age ≥ 75 years (odds ratio [OR] = 2.151; 95% confidence interval [CI], 1.414–3.272; *p* < 0.001), severe osteoporosis (OR = 2.824; 95% CI, 1.481–5.381; *p* = 0.002), rigid lumbar curve with dynamic changes in LL < 10° (OR = 5.150; 95% CI, 2.296–11.548; *p* < 0.001), and severe facet joint osteoarthritis (OR = 4.513; 95% CI, 1.958–10.402; *p* < 0.001) were independent risk factors for PI − LL mismatch ≥ 10° after A-P surgery.

**Conclusion:**

P-A-P sequence for deformity corrective surgery in ASD offers greater LL correction than the A-P sequence. Indications for the procedure include patients aged ≥ 75 years, severe osteoporosis, rigid lumbar curve with dynamic change in LL < 10°, or more than four facet joints of Pathria grade 3 in the lumbar region.

## Background

Restoration of successful and harmonious overall spinopelvic alignment has become a key consideration in the surgical treatment of adult spinal deformity (ASD). It has been demonstrated that the maintenance and restoration of global sagittal balance are critical to the quality of life and improvement in function following spinal surgery since optimal sagittal alignment reduces compressive forces on vertebral bodies and intervertebral discs and muscular energy expenditure, improves spinal biomechanical efficiency, and decelerates adjacent segment degeneration [1–6].

Various osteotomy techniques, such as Smith–Peterson osteotomy and Ponte osteotomy, have been developed to address sagittal deformities. These two procedures are the most commonly used posterior-column-only osteotomy techniques that can be used at multiple levels with a low risk of complications [7, 8]. For patients with fixed sagittal imbalance, pedicle subtraction osteotomy has been used more frequently to obtain greater correction with a single posterior approach [9]. Unfortunately, this procedure presents technical challenges with significant morbidity and may be associated with a high incidence of complications such as pseudarthrosis, rod breakage, and severe bleeding [10–12]. Also, overcorrection at a single level may make it difficult to achieve physiological lordosis, leading to disruption of spine biomechanics [13].

With ongoing advancements in surgical techniques and instruments, anterior column realignment (ACR) using retroperitoneal lateral lumbar interbody fusion (LLIF), [14–16] combined with posterior osteotomy, has been widely adopted for the correction of spinal deformities [15–17]. LLIF is a relatively new technique that can be performed via the anterior or trans-psoas approach, allowing the surgeon to access the disc space [14, 18, 19]. In general, ACR is performed first in combined anterior-posterior (A-P) surgery. Following complete discectomy, intentional release of the anterior longitudinal ligament (ALL) is performed in conjunction with the placement of a hyperlordotic interbody cage with a wide footprint for greater segmental correction. Posterior instrumentation and fusion with multiple osteotomies are then subsequently performed [20–23]. Several studies have demonstrated that posterior-only surgery is inferior to combined A-P surgery due to decreased deformity correction, increased pseudarthrosis rates, and persistent sagittal imbalance. In addition, combined approach surgery is believed to be more effective in restoring segmental lordosis (SL) and reducing the risk of adjacent segment disease [24, 25].

Although this traditional combined A-P surgeries are effective in most patients with ASD, several studies have demonstrated that cage subsidence is a potentially devastating complication after spinal surgery, especially during the early period when successful fusion has not been achieved [20, 26]. Furthermore, the A-P procedure has been reported to result in suboptimal sagittal correction for certain severe rigid spinal deformities [20, 26]. In such patients, a purely traditional A-P surgery does not provide adequate sagittal correction due to a progressive loss in SL and foraminal height resulting from cage subsidence.

To our knowledge, there is a lack of research regarding the effect of posterior osteotomy prior to ACR on the surgical correction for spinal deformities [27]. Moreover, the optimal procedure for patients with a fixed sagittal imbalance remains controversial. For these reasons, the author hypothesized that posterior osteotomy prior to ACR through P-A-P surgical sequence would offer greater lordosis correction than the conventional A-P sequence for patients with ASD. Therefore, this study aimed to determine the impact of the P-A-P sequence on the restoration of lumbar lordosis (LL) compared to the A-P sequence in deformity corrective surgery for ASD patients and to identify the characteristics of patients who require this sequence.

## Methods

### Patient recruitment

This was a retrospective, single institution, case-control study of consecutive patients with ASD who underwent corrective surgery for spinal deformities between January 2017 and December 2019. This study included the patients aged 60 years or older who had a primary diagnosis of ASD with sagittal imbalance, which was defined by at least one of the following radiographic measurements: C7 sagittal vertical axis (C7SVA) ≥ 100 mm, pelvic tilt (PT) ≥ 25°, or pelvic incidence (PI) minus LL mismatch (PI − LL mismatch) ≥ 10°. Other inclusion criteria were as follows: (1) posterior spinal fusion to the sacrum (≥ 5 vertebrae) using the pedicle screw system, (2) selective LLIF at L1–5 levels (≥ 2 levels), and (3) more than two-year follow-up periods. Patients with a history of spinal trauma, infection, or tumors were excluded from this study. All patients failed at least six months of conservative management before surgery. The patients were divided into two groups according to the surgical sequence used. From January 2017 to March 2018, the traditional A-P sequence was routinely performed on the first part of the patients (A-P group). However, from April 2018 to December 2019, the P-A-P sequence was conducted to the second part of patients (P-A group) preferentially because the author was aware of the improved clinical and radiographic outcomes using this technique. All procedures were performed in a staged fashion with motor-evoked potential monitoring by a single surgeon.

### Data collection

The clinical and radiological data of the patients were obtained by reviewing the medical records and the picture archiving communication system (PACS) of our institution. The baseline assessment consisted of standard demographics, including age, sex, body mass index (BMI), preoperative diagnosis (indication for fusion), history of prior spine surgery, Charlson comorbidity index (CCI), and the American Society of Anesthesiologists (ASA) classification. All patients underwent at least one dual-energy X-ray absorptiometry scan of the lumbar spine to measure bone mineral density (BMD). In this study, severe osteoporosis was defined as one or more fragility fractures in patients with a T-score ≤ − 2.5.[28].

Several variables pertinent to the operative data were recorded for each patient, including the type of approach, number of levels fused, level of laminectomy, upper instrumented vertebra (UIV) level, operative time, estimated blood loss (EBL), method for interbody fusion at L5-S1, and length of hospital stay. Major complications, such as postoperative neurological deterioration, surgical site infection, or other instrument-related complications, were also analyzed in detail. Proximal junctional kyphosis (PJK) was defined as an absolute proximal junctional angle (PJA) > 10° or an increase in PJA > 10° compared to preoperative measurements, with the PJA being measured from the inferior endplate of the UIV to the superior endplate of the vertebra two levels above the UIV [2]. Pseudarthrosis was defined as the lack of solid bony growth across the disc space or facet at least one year after surgery on either plain films or computed tomography (CT) scans and the presence of motion on flexion-extension radiographs [29]. Cage subsidence was evaluated using multiplanar reconstructed CT images, which were defined as the sinking of the interbody cage by more than 2 mm into the adjacent vertebral bodies [26].

### Radiographic assessment

Whole-spine lateral radiographs were analyzed preoperatively and two years postoperatively, with the patient standing in a neutral unsupported fists-on-clavicle position [30]. The following spinopelvic radiographic parameters were measured according to previously reported methods: [31, 32] C7SVA, the distance from the C7 plumb line to a perpendicular line drawn from the posterosuperior corner of the S1; thoracic kyphosis (TK), the angle between the superior endplate of T5 and the inferior endplate of T12; thoracolumbar kyphosis (TLK), the angle between the superior endplate of T10 and the inferior endplate of L2; PT, the angle between the vertical and the line drawn through the sacral endplate midpoint to the femoral head axis; PI, the angle between the line drawn from the femoral head axis to the midpoint of the sacral endplate and the line perpendicular to the sacral endplate; LL, the angle between the superior endplate S1 and the superior endplate of L1. To measure SL, tangent lines were drawn along the inferior endplate of the superior vertebral body, and the superior endplate of the inferior vertebra at the level of interest, and the angle formed by the intersection of the two lines was SL [33, 34]. Finally, PI − LL mismatch was also calculated, which was generally considered a predictor of ideal sagittal alignment following reconstructive surgery [4, 35]. With all measurements, angles were noted as positive (+) if kyphotic and negative (−) if lordotic.

As described in a previous publication, [34, 36] the flexibility of the lumbar spine was evaluated based on the baseline dynamic LL angle, which was defined as the difference in LL between lateral dynamic flexion-extension radiographs. This study generally defined a rigid lumbar curve as a dynamic change in LL < 10°.

Preoperative and two-year postoperative spinopelvic radiographic parameters were independently collected by two spine surgeons who were not involved in the operative treatment. The inter-rater and intra-rater reliabilities were calculated using kappa statistics. The same two spine surgeons measured the data mentioned above for a second time, with an interval of two weeks. The intraclass correlation coefficient (ICC) was measured to assess agreement between observers [37, 38].

### Assessment of facet joint osteoarthritis

Radiographic assessment of preoperative facet joint degenerative osteoarthritis (OA) was conducted based on the criteria proposed by Pathria et al. [39] The severity of facet joint OA on CT was classified into the following four grades: G0, normal; G1, facet joint narrowing; G2, facet joint narrowing with sclerosis or hypertrophy; and G3, severe arthritis with facet joint narrowing, sclerosis, and osteophytes [39]. This study defined severe facet joint OA as > 4 Pathria G3 facet joints at L2–5 levels (range, 0–8).

### Surgical procedures

#### P-A-P sequence: posterior osteotomy prior to ACR

In general, the P-A-P surgical sequence is as follows: First, the patients were carefully placed prone, a standard midline incision was made with fluoroscopic confirmation of the operation level, and the paraspinal muscles were detached. Subperiosteal dissection was performed to expose the spinous processes, laminae, facet joints, and transverse processes at the cephalad and caudal levels. Before bone decompression, bilateral pedicle screws were inserted at the index level. Subsequently, extensive posterior spinal release was performed using elective multilevel inferior facetectomy to gain flexibility for correctional maneuvers. Decompressive laminectomy was performed in the patients with symptomatic lumbar spinal stenosis. The authors generally prefer no resection of the cephalad portion of the lamina. If necessary, partial resection of the tip of the superior articular process (SAP) was performed in patients with combined lumbar foraminal stenosis. Lumbar interbody fusion was performed at the L5-S1 level when necessary. The bone obtained from the posterior elements was preserved for autogenous bone grafting in the ACR.

One week later, the author performed selective LLIF through a lateral retroperitoneal approach, as previously described [14–19]. Initially, the patients were positioned in a left lateral decubitus position. Through an oblique skin incision, the retroperitoneal space was entered by blunt dissection with fingers or sponges to expose the iliopsoas muscle and lumbar spine. After confirming the target level with fluoroscopy, followed by excision of the annulus fibrosus, the nucleus pulposus, and cartilaginous endplate were circumferentially resected carefully to avoid vertebral endplate injury. In addition, electrocautery was avoided, and a #15 scalpel blade was used to cut the ALL to minimize damage to the surrounding tissues while preserving one-third of the ligament. Next, a hyperlordotic interbody cage was inserted, which was determined intraoperatively at the relevant disc level by inserting sequential trials. All cages were filled with autogenous bone from the posterior bony elements, and the intervertebral disc space was filled with a mixture of chipped-bone allograft and demineralized bone matrix to enhance the fusion rate. After confirmation of appropriate cage placement using fluoroscopy and meticulous hemostasis, a drainage tube was placed, and the fascia, subcutaneous layer, and skin were sutured.

Finally, the patients are prone to intraoperative repositioning. Proper pre-contoured rods were selected and used to lock the assembly into the screw heads. Spinal deformity correction was mainly performed by postural correction on the operating table using the cantilever bending technique.

#### **A-P sequence: no posterior osteotomy prior to ACR**

The ACR procedure was performed in the first stage, as described for the P-A-P sequence. One week later, elective facetectomy and laminectomy with posterior instrumentation were performed, as described in our P-A-P sequence. Finally, rods of appropriate length were chosen and contoured properly to achieve the target lumbar curvature.

### Statistical analysis

All statistical analyses were performed using SPSS software (IBM SPSS Statistics, Version 21.0; IBM Corp., Armonk, NY, USA). All continuous variables are presented as the mean ± standard deviation and compared using a t-test between the two independent groups and paired t-test within each group. For categorical variables, the number and proportion of each modality were calculated and compared using Pearson’s chi-square test. Ordinal variables were compared between the two independent groups using the Mann-Whitney U test. Significant variables in univariate analyses were evaluated using a multivariate logistic regression analysis to identify the risk factors for postoperative PI − LL mismatch ≥ 10°. A *p*-value of 0.05 or lower was considered statistically significant.

## Results

### Baseline demographic data

A total of 260 patients with a mean T-score of -1.87 were enrolled (Table 1). Of these, 93 (36%) had undergone prior spinal surgery. The P-A group included 150 females and 28 males with a mean age of 73.6 years and a mean BMI of 25.6 kg/m^2^. The A-P group included 68 females and 14 males, with a mean age of 72.4 years and a mean BMI of 26.7 kg/m^2^. Both groups were similar in terms of age, sex, BMD, preoperative diagnosis, dominant symptoms, CCI, and ASA scores (*p* > 0.05).

**Table 1:** Patient demographics data of the P-A and A-P groups

Variable	P-A group	A-P group	*p*-value
Number of patients	178	82	
Age, years	73.6 ± 5.6	72.4 ± 7.2	0.168
Sex, (F/M)	150/28	68/14	0.785
BMI, kg/m^2^	25.6 ± 5.15	26.7 ± 5.4	0.114
BMD, T-score	-1.9 ± 1.1	-1.9 ± 1.5	0.680
Osteoporosis/osteopenia, n (%)	82 (46.1)	44 (53.7)	0.255
Revision surgery, n (%)	65 (36.5)	28 (34.1)	0.711
Dominant symptoms, n (%)			
Back pain	142 (79.8)	70 (85.4)	0.280
Leg pain	129 (72.5)	63 (76.8)	0.458
Claudication	97 (54.5)	43 (52.4)	0.757
Numbness/tingling	68 (38.2)	30 (36.6)	0.803
Weakness	29 (16.3)	14 (17.1)	0.875
CCI	2.4 ± 1.2	2.6 ± 1.4	0.082
Preoperative diagnosis, n (%)			0.784
Adult idiopathic scoliosis	43 (24.2)	21 (25.6)	
Degenerative scoliosis	37 (20.8)	14 (17.1)	
Degenerative sagittal imbalance	76 (42.7)	39 (47.6)	
Postoperative state of lumbar spine	22 (12.4)	8 (9.8)	
ASA class, n (%)			0.908
I	29 (16.3)	14 (17.1)	
II	129 (72.5)	58 (70.7)	
III	20 (11.2)	10 (12.2)	

Bold text indicates statistical significance (*p* < 0.05)

*BMI* body mass index, *BMD* bone mineral density, *CCI* Charlson comorbidity index, *ASA* American Society of Anesthesiologists

### Comparison of the operative details and complications

The operative details of each group are summarized in Table 2. Overall, the mean number of levels fused was 7.58 ± 0.6, and pelvic fixation with iliac screws was used in 179 patients (68.8%). The mean number of levels decompressed was 3.2 ± 1.9 for the P-A group, whereas 3.5 ± 1.3 for the A-P group. The distribution of the LLIF levels was similar between the groups. The patients in P-A group had a significantly longer operation time (347.2 min vs. 330.4 min, *p* = 0.016) and more EBL (2.3 L vs. 2.0 L, *p* = 0.028) than patients in A-P group. Nevertheless, there were no differences in the length of stay and the number of patients that required intensive care between the groups.

**Table 2:** Operative details and complications between the P-A and A-P groups

Variable	P-A group	A-P group	*p*-value
Number of patients	178	82	
Number of levels fused	7.6 ± 0.7	7.5 ± 0.3	0.535
Number of levels decompressed	3.2 ± 1.9	3.5 ± 1.3	0.196
Number of LLIF	3.5 ± 0.4	3.4 ± 0.5	0.142
LLIF level, n (%)			
L1/2	6 (3.4)	5 (6.1)	0.494
L2/3	150 (84.3)	75 (91.5)	0.114
L3/4	162 (91.0)	78 (95.1)	0.166
L4/5	169 (94.9)	79 (96.3)	0.856
UIV, n (%)			0.725
T9-T10	142 (79.8)	63 (76.8)	
T11-T12	20 (11.2)	9 (11.0)	
L1-L2	16 (9.0)	10 (12.2)	
Interbody fusion at L5/S1, n (%)			0.418
PLIF	141 (79.2)	58 (70.7)	
TLIF	22 (12.4)	12 (14.6)	
None	10 (5.6)	8 (9.8)	
Previous fusion	5 (2.8)	4 (4.9)	
Sacropelvic fixation, n (%)	123 (69.1)	56 (68.3)	0.896
Operation time, (min)	347.2 ± 53.1	330.4 ± 48.7	**0.016**
EBL, (L)	2.3 ± 1.1	2.0 ± 0.8	**0.028**
Length of hospital stay, (day)	30.7 ± 7.2	29.2 ± 7.3	0.121
Number of ICU stay, n (%)	7 (3.9)	2 (4.9)	0.805
Complication, n (%)			
PJK	11 (6.2)	8 (9.8)	0.303
Pseudarthrosis	5 (2.8)	4 (4.9)	0.629
Cage subsidence	17 (9.6)	21 (25.6)	**0.001**
Neurologic deficit	6 (3.4)	6 (7.3)	0.275
Dural tear	12 (6.7)	6 (7.3)	0.865
Deep vein thrombosis	2 (1.7)	2 (2.4)	0.796
Pneumonia	5 (2.8)	1 (2.4)	0.727
Surgical site infection	4 (2.2)	1 (2.4)	0.940

Bold text indicates statistical significance (*p* < 0.05)

*LLIF* lateral lumbar interbody fusion, *UIV* upper instrumented vertebrae, *PLIF* posterior lumbar interbody fusion, *TLIF* transforaminal lumbar interbody fusion, *EBL* estimated blood loss, *ICU* intensive care unit, *PJK* proximal junctional kyphosis

Postoperative complications were assessed. The occurrence of cage subsidence was significantly higher in the A-P group than in the P-A group (25.6% vs. 9.6%, *p* = 0.001). PJK occurred in 11 patients (6.2%) in the P-A group and eight patients (9.8%) in the A-P group, showing no significant difference between the two groups. In addition, the pseudarthrosis rate was not significantly different between the two groups (P-A, 2.8%; A-P, 4.9%; *p* = 0.629). Nevertheless, no patients underwent additional revision surgery because of the absence of clinical symptoms. There were two cases of neurological deterioration secondary to foraminal narrowing after ACR in the A-P group. No significant differences in the incidence of transient neurological deficits, deep vein thrombosis, pneumonia, or surgical site infection were found between the two groups. All complications were resolved by the time of discharge.

### Comparison of the preoperative and postoperative sagittal spinopelvic parameters

The radiographic sagittal spinopelvic parameters are summarized in Table 3. There were no significant differences in the sagittal spinopelvic parameters before surgery between the groups. The mean LL in the P-A and A-P groups were − 3.5° and − 4.0° preoperatively, -57.2° and − 48.3° at the final follow-up, respectively, representing statistically significant differences within groups from the preoperative values (*p* < 0.05). Similarly, the mean C7SVA was 212.5 mm in the P-A group and 184.9 mm in the A-P group before surgery, which was significantly improved at two years postoperatively compared to the preoperative values in both groups. In addition, both groups showed significant improvements in TK, TLK, PT, and the corresponding SL after the surgery. Some sagittal spinopelvic parameters, such as C7SVA, LL, and SL (L2-3, L3-4, and L4-5), were significantly higher in the P-A group at the final follow-up than in the A-P group (*p* < 0.05).

**Table 3:** Preoperative and postoperative radiographic parameters between the P-A and A-P groups

Parameter		P-A group	A-P group	*p*-value
Number of patients		178	82	
C7 SVA	Preoperative	212.5 ± 195.3	184.9 ± 178.2	0.278
	Postoperative	15.2 ± 35.3	38.8 ± 58.4	** < 0.001**
	Change	-197.4 ± 178.3	-146.1 ± 135.5	**0.021**
TK	Preoperative	11.6 ± 10.7	13.1 ± 12.6	0.333
	Postoperative	27.8 ± 18.3	28.9 ± 15.8	0.663
	Change	16.3 ± 5.5	15.8 ± 6.2	0.556
TLK	Preoperative	32.7 ± 21.5	29.4 ± 17.4	0.228
	Postoperative	14.9 ± 6.6	16.2 ± 9.3	0.188
	Change	-17.8 ± 15.7	-13.2 ± 13.5	**0.023**
PI	Preoperative	60.0 ± 10.8	61.7 ± 14.0	0.283
PT	Preoperative	38.4 ± 17.4	34.9 ± 14.3	0.106
	Postoperative	21.0 ± 4.2	20.3 ± 8.7	0.390
	Change	-17.4 ± 18.0	-14.6 ± 10.2	0.177
LL	Preoperative	-3.5 ± 5.7	-4.0 ± 5.4	0.523
	Postoperative	-57.2 ± 25.8	-48.3 ± 12.8	**0.004**
	Change	-53.7 ± 32.5	-44.3 ± 19.8	** < 0.001**
PI − LL	Preoperative	56.5 ± 31.2	57.7 ± 27.9	0.761
	Postoperative	2.9 ± 5.5	13.4 ± 8.4	** < 0.001**
Number of patients		6	5	
SL L1/2	Preoperative	1.5 ± 0.8	1.5 ± 0.8	0.919
	Postoperative	-3.4 ± 1.4	-3.5 ± 1.5	0.937
	Change	-4.9 ± 1.7	-5.0 ± 1.9	0.929
Number of patients		150	75	
SL L2/3	Preoperative	1.3 ± 0.3	1.4 ± 0.3	0.127
	Postoperative	-14.9 ± 6.2	-13.0 ± 7.2	**0.041**
	Change	-16.2 ± 5.8	-14.4 ± 7.1	**0.043**
Number of patients		162	78	
SL L3/4	Preoperative	1.6 ± 0.7	1.5 ± 0.2	0.151
	Postoperative	-14.6 ± 5.2	-12.3 ± 6.4	**0.003**
	Change	-16.2 ± 5.8	-13.8 ± 6.6	**0.004**
Number of patients		169	79	
SL L4/5	Preoperative	-1.9 ± 0.9	-2.1 ± 1.3	0.187
	Postoperative	-16.9 ± 7.3	-13.4 ± 6.6	** < 0.001**
	Change	-15.0 ± 8.5	-11.3 ± 8.2	**0.001**

Bold text indicates statistical significance (*p* < 0.05)

All parameters are in degrees (◦) except SVA (mm)

*SVA* sagittal vertical axis, *TK* thoracic kyphosis, *TLK* thoracolumbar kyphosis, *PI* pelvic incidence, *PT* pelvic tilt, *LL* lumbar lordosis, *SL* segmental lordosis

Regarding the change of sagittal spinopelvic parameters, the P-A group had a significantly greater amount of the changes in C7SVA (-197.4 ± 178.3 mm vs. -146.1 ± 135.5 mm, *p* = 0.023), LL (-53.6 ± 32.5° vs. -44.3 ± 19.8°, *p* = 0.017), and TLK (-17.8 ± 15.7° vs. -13.2 ± 13.5°, *p* = 0.023) than the A-P group. In addition, significant differences were found between the P-A and A-P groups in the restoration of SL L2-3 (-16.2° vs. -14.4°, *p* = 0.043), SL L3-4 (-16.2° vs. -13.8°, *p* = 0.004), and SL L4-5 (-15.1° vs. -11.3°, *p* = 0.001), respectively. Changes in other sagittal spinopelvic parameters were similar between the groups. At the final follow-up, PI − LL mismatch was significantly higher in the A-P group (13.4° vs. 2.9°, *p* < 0.001). In the P-A group, all 178 patients had postoperative ideal sagittal alignment, compared to 75.6% (62/82) in the A-P group.

### Predictive risk factors for PI − LL mismatch ≥ 10°

According to Schwab et al. [4] optimal sagittal alignment was defined as PI − LL mismatch < 10°, which is a critical radiological parameter strongly related to patient-reported outcomes (PRO). For further analysis, the A-P group was divided into two subgroups according to a postoperative PI − LL mismatch threshold of 10°. Sixty-two patients (52 females and ten males) were included in the PI − LL mismatch < 10° group, and 20 patients (16 females and four males) were included in the PI − LL mismatch ≥ 10° group. As shown in Table 4, significant differences were observed between the subgroups in terms of BMD and the distribution of elderly aged ≥ 75 years, [40] severe osteoporosis, severe facet joint OA, and rigid lumbar curve with dynamic changes in LL < 10°. Significant variables in univariate analyses were examined to determine the risk factors for developing a postoperative PI − LL mismatch ≥ 10° using stepwise logistic regression analysis. Factors with a *p*-value < 0.10 in univariate analysis were included in the multivariate analysis. Among these variables, age ≥ 75 years (odds ratio [OR] = 2.151; 95% confidence interval [CI], 1.414–3.272; *p < 0.001*), severe osteoporosis (OR = 2.824; 95% CI, 1.481–5.381; *p = 0.002*), rigid lumbar curve with dynamic changes in LL < 10° (OR = 5.150; 95% CI, 2.296–11.548; *p < 0.001*), and severe facet joint osteoarthritis (OR = 4.513; 95% CI, 1.958–10.402; *p < 0.001*) significantly increased the probability of developing postoperative PI − LL mismatch ≥ 10° in the A-P group (Table 5).

**Table 4:** Data for patients that underwent the A-P procedure grouped by PI-LL mismatch threshold of 10°

Characteristics	All	PI-LL mismatch < 10° group	PI-LL mismatch ≥ 10° group	*p*-value
Number of patients	82	62	20	
Age, (≥ 75 years/ < 75 years)	46/36	30/32	16/4	**0.013**
Sex, (F/M)	68/14	52/10	16/4	0.953
BMI, kg/m^2^	26.7 ± 5.4	26.5 ± 4.9	27.5 ± 5.7	0.419
BMD, T-score	-1.9 ± 1.5	-1.7 ± 1.2	-2.7 ± 1.7	**0.005**
Severe osteoporosis, n (%)	38	22 (35.5)	16 (80.0)	**0.001**
CCI	2.7 ± 1.4	2.6 ± 1.2	2.8 ± 1.5	0.531
UIV, n (%)				0.446
T9-T10	63	49	14	
T11-T12	9	7	2	
L1-L2	10	6	4	
Number of levels fused	7.6 ± 0.3	7.5 ± 0.3	7.6 ± 0.8	0.396
Number of LLIF	3.4 ± 0.5	3.3 ± 0.5	3.5 ± 0.6	0.164
Number of levels laminectomy	3.5 ± 1.3	3.5 ± 1.2	3.4 ± 1.6	0.722
Severe facet joint OA, n (%)	45	30 (48.4)	15 (75.0)	**0.038**
Dynamic changes in LL < 10°, n (%)	32	20 (32.3)	14 (70.0)	**0.003**

Bold text indicates statistical significance (*p* < 0.05)

*BMI* body mass index, *BMD* bone mineral density, *CCI* Charlson Comorbidity Index, *UIV* upper instrumented vertebrae, *LLIF* lateral lumbar interbody fusion *OA* osteoarthritis, *PI* pelvic incidence, *LL* lumbar lordosis

**Table 5:** Potential risk factors for developing postoperative PI-LL mismatch ≥ 10°(Multivariate logistic regression analysis)

	Regression Coefficient	Standard Error	Waldχ^2^	*p*-value	OR	95% CI
Age ≥ 75 years	0.766	0.214	12.812	** < 0.001**	2.151	1.414–3.272
Severe osteoporosis	1.038	0.329	9.954	**0.002**	2.824	1.481–5.381
Severe facet joint OA	1.507	0.426	12.514	** < 0.001**	4.513	1.958–10.402
Dynamic changes in LL < 10°	1.639	0.412	15.826	** < 0.001**	5.150	2.296–11.548
Constant	-1.921	0.437	19.323	< 0.001	0.146	

Bold text indicates statistical significance (*p* < 0.05)

*OA* osteoarthritis, *PI* pelvic incidence, *LL* lumbar lordosis

### Assessment of the reliability of radiographic measurements using ICC

The ICC values for all radiographic measurements showed good to excellent inter-rater and intra-rater reliabilities. The ICC for intra-rater reliability was good to excellent (0.82 to 0.97) for the measurements. The intra-rater reliability of the preoperative measurements (0.88 to 0.97) was good or excellent and somewhat better than that of two-year postoperative measurements (0.82 to 0.95). The ICC for the inter-rater reliability of the radiographic measurements was also good or excellent (0.79 to 0.95). In general, preoperative measurements (0.81 to 0.95) tended to have higher reliability than the two-year postoperative measurements (0.79 to 0.93). Moreover, the second measurement (0.82 to 0.95) was more reliable than the first measurement (0.79 to 0.91).

### Illustrative cases

#### Patient 1

**Fig. 1:**
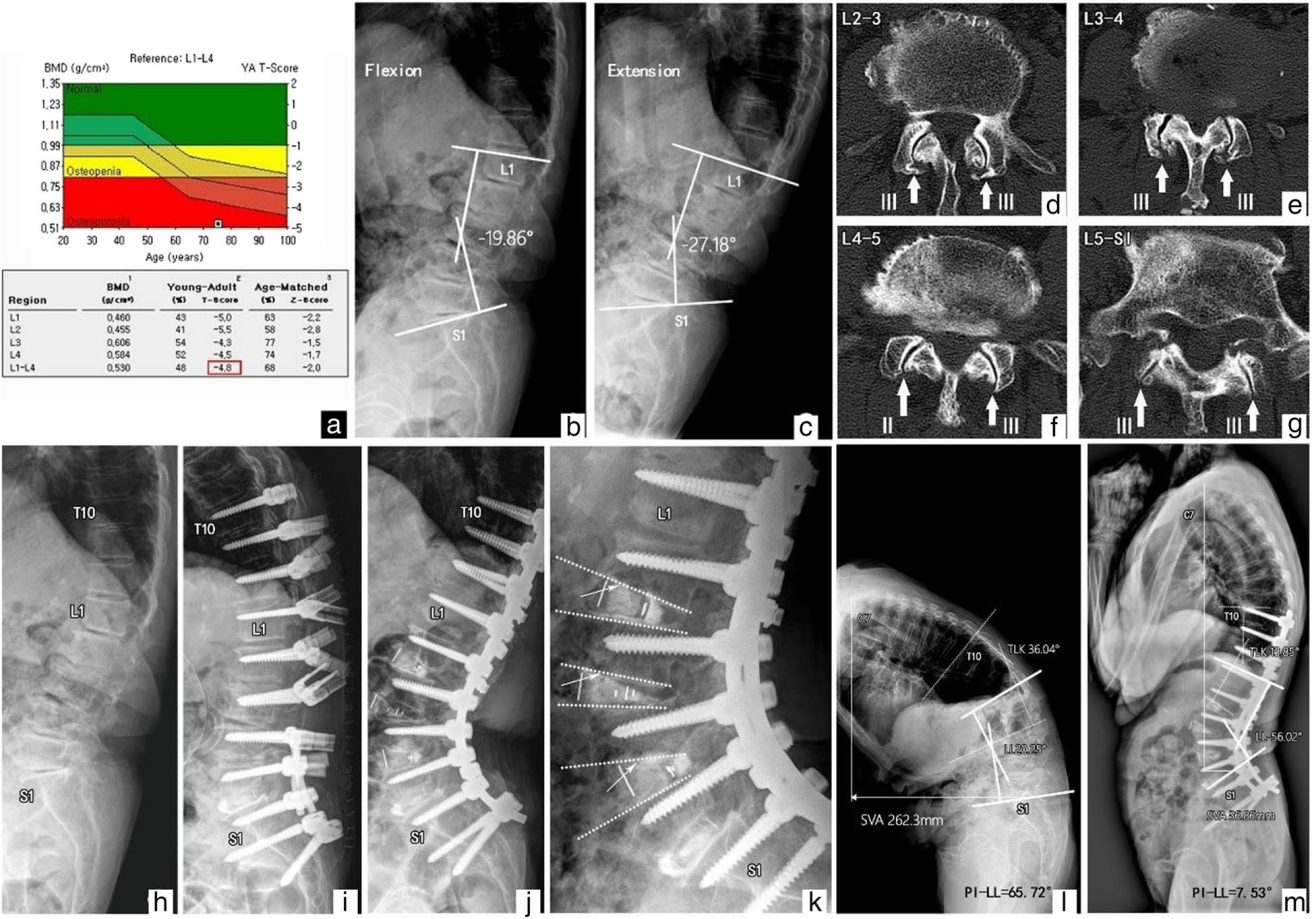
Example of a patient who underwent deformity correction surgery using the P-A-P procedure; (**a**) T-score of -4.8; (**l**) The sagittal spinopelvic parameters on preoperative whole-spine X-ray were as follows: SVA, 262.3 mm; LL, 20.25°; TLK, 36.04°; PI, 45.47°; and PI − LL mismatch, 65.72°; (**b**, **c**) The measurements of LL on flexion and extension radiographs were − 19.86° and − 27.18°, respectively; (**d-g**, thick arrow) Computed tomography (CT) illustrated the grades of facet joint osteoarthritis using the Pathria grading scale as follows: (III-III), (III-III), (II-III), (III-III), respectively; (**h-k**) Index segmental lordosis significantly increased after surgery, and cage subsidence was not observed; (**m**) Postoperative two-year X-ray showed a well-maintained optimal sagittal alignment (SVA, 36.86 mm; LL, -56.02°; TLK, 14.66°; PI, 48.49°; and PI − LL mismatch, 7.53°)

A 73-year-old female with a T-score of -4.8 underwent deformity correction surgery using the P-A-P sequence due to progressive lower back and leg pain. Before surgery, the sagittal spinopelvic parameters on the whole-spine radiograph were as follows: C7SVA, 262.3 mm; LL, 20.25°; TLK, 36.04°; PI, 45.47°; and PI − LL mismatch, 65.72°. The measurements of LL on flexion and extension radiographs were − 19.86° and − 27.18°, respectively. CT revealed the grades of facet joint OA using the Pathria grading scale as follows: (L2-3:3–3), (L3-4:3–3), (L4-5:2–3), and (L5-S1:3–3), respectively. She underwent LLIF at the L2–5 levels after posterior osteotomies. SL at the index levels increased significantly immediately after surgery, and no cage subsidence occurred. At the final follow-up, satisfactory global sagittal alignment was maintained, and the C7SVA, LL, TLK, PI, and PI − LL mismatch were 36.86 mm, -56.02°, 14.66°, 48.49°, and 7.53°, respectively (Fig. 1).

#### Patient 2

**Fig. 2:**
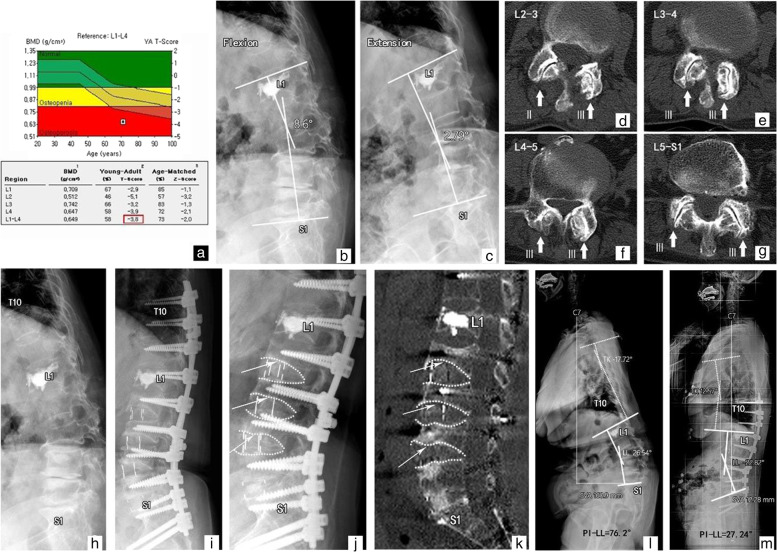
Example of a patient who underwent deformity correction surgery using the A-P procedure; (**a**) T-score of -3.8; (**l**) The sagittal spinopelvic parameters on preoperative whole-spine X-ray were as follows: SVA, 160.9 mm; LL, 26.54°; TK, -17.72°; PI, 49.66°; and PI-LL mismatch, 76.2°; (**b**,** c**) The measurements of LL on flexion and extension radiographs were 8.6° and 2.79°, respectively.; (**d-g**, thick arrow) Computed tomography (CT) illustrated the severity of facet joint osteoarthritis based on the Pathria grading system as follows: (II-III), (III - III), (III - III), (III - III), respectively; (**h-k **thin arrow) Index segmental lordosis slightly increased after surgery, and cage subsidence was observed at operated levels; (**m**) Postoperative two-year X-ray showed an optimal sagittal balance with an unsatisfactory improvement of LL (SVA, 12.28 mm; LL, -22.82°; TK, 12.57°; PI, 50.06°; and PI-LL mismatch, 27.24°)

A 77-year-old female with a T-score of -3.8 underwent deformity correction surgery using the A-P procedure due to neurogenic claudication and difficulty walking. Before surgery, the sagittal spinopelvic parameters on the whole-spine radiograph were as follows: C7SVA, 160.9 mm; LL, 26.54°; TK, -17.72°; PI, 49.66°; and PI − LL mismatch, 76.2°. The measurements of LL on flexion and extension radiographs were 8.6° and 2.79°, respectively. CT revealed the grades of facet joint OA using the Pathria grading scale as follows: (L2-3:2–3), (L3-4:3–3), (L4-5:3–3), and (L5-S1:3–3), respectively. She underwent LLIF at the L2–5 levels without posterior osteotomies prior to ACR. The SL at the index levels increased immediately after surgery, whereas cage subsidence and vertebral collapse were significantly observed at the operated levels. At the final follow-up, a good sagittal balance with an unsatisfactory improvement of LL was maintained, and the C7SVA, LL, TLK, PI, and PI − LL mismatch were 12.28 mm, -22.82°, 12.57°, 50.06°, and 27.24°, respectively (Fig. 2).

## Discussion

Numerous studies have been conducted to understand the importance of sagittal alignment in ASD surgery, and it is widely accepted that restoration of adequate LL and correction of PI − LL mismatch prevents sagittal decompensation after reconstructive spinal surgery [1, 41, 42]. Thus, restoring optimal LL and sagittal alignment has become an important parameter for improved long-term clinical outcomes of deformity correction surgeries.

Cage subsidence is the most common perioperative complication after interbody fusion and usually occurs under compressive loading at the cage-endplate interface, leading to progressive loss of SL, disc height, and foraminal dimension [26, 43]. It is well known that the potential causes of cage subsidence are multifactorial, ranging from patient characteristics and surgical risk factors to implant materials properties [44–46]. In this study, patients treated with the P-A-P sequence had significantly less cage subsidence than those treated with the A-P sequence. Interestingly, all the patients had similar demographic characteristics, comorbidities, and spinopelvic parameters before surgery. Regarding the implant material, all patients received interbody fusion using the polyetheretherketone (PEEK) cage. Cage subsidence was observed in both groups, which seemed unavoidable. Nevertheless, this finding may suggest that by modifying the traditional A-P sequence, which involves the addition of posterior spinal osteotomy prior to ACR, even less cage subsidence can be achieved. In other words, the inadequate release of posterior elements can increase intervertebral compression stress during LLIF, which may further increase the possibility of cage subsidence. It is worth noting that most cage subsidence in the A-P group was observed during ACR steps. Considering that LLIF was performed in the first stage, the author provided evidence to suggest that patients treated with posterior spinal osteotomy prior to ACR had significantly less cage subsidence than those treated with traditional combined A-P surgery.

To the best of our knowledge, this is the first study to show that posterior osteotomy before ACR may reduce the incidence of cage subsidence in patients with ASD. Regarding the A-P sequence, concerns remain regarding whether sufficient LL correction via ACR can be achieved without vertebral endplate injury in patients with severe sagittal imbalance. In particular, most cage subsidence occurred during LLIF in the AP group. Given that posterior spinal release was performed in the first stage of the P-A-P sequence, the author believed that the disparity in the incidence of cage subsidence was due to compressive stress from posterior spinal elements during LLIF. The lower cage subsidence rate of the P-A group might be explained by the fact that we performed posterior releases prior to ACR, which reduced the mechanical compressive strength of the cage-endplate interface. Hence, posterior spinal release prior to ACR is necessary for patients with a higher risk of cage subsidence.

Posterior column osteotomy has been well described and was originally reported to shorten the posterior column for sagittal correction by stepwise resection of the posterior ligamentous complex, spinous processes, facet joints, and lamina, which may also improve axial flexibility of the spinal segment. Oda et al. [47] previously reported that complete facetectomy and posterior spinal release could provide an approximately 45% increase in axial rotation of the spine with uniformly applied torque. Wiemann et al. [48] demonstrated that Smith–Peterson and Ponte osteotomy decreased the force required to rotate spinal segments concerning the axial plane by approximately one-fifth.

Generally, indirect ALL release with partial discectomy at multiple levels appears to provide adequate release of the anterior and middle columns. However, as evidenced by our results, achieving adequate LL using the A-P sequence may be difficult in some circumstances. Facet joints are complicated biomechanical structures located at the back of the spine. Facet joint OA is common in older adults and has classic radiographic characteristics of arthritis, including non-uniform joint space loss, subarticular bone erosion, cyst formation, facet hypertrophy, and osteophyte formation. Stiffness and decreased range of motion (ROM) are common symptoms of severe facet joint OA [49, 50]. Joint flexibility is well known to decrease with age [51]. As described by Fujiwara et al. [52], facet joint OA is likely to limit segmental motion. Thus, spinal flexibility is affected by facet joint OA. This study indicated that posterior spinal release prior to ACR might offer better spinal flexibility, which allows for more distraction of the intervertebral disc space. Schulte et al. [53] reported that a significant increase in ROM was observed in flexion-extension movements after facetectomy. This may explain why patients in the A-P group had a higher likelihood of relatively insufficient correction in the current study. Consequently, posterior spinal release via posterior osteotomy prior to ACR to increase the flexibility of the spinal segment is thought to be necessary for severe rigid deformity.

In recent years, sagittal spinopelvic alignment has attracted considerable interest among spinal surgeons. Several studies have demonstrated that appropriate restoration of spinal alignment can lead to significant improvements in pain associated with radiculopathy, neurogenic claudication, segmental deformity, or instability [54–56]. Additionally, it is widely accepted that postoperative sagittal malalignment is a risk factor for poor clinical outcomes and junctional kyphosis [57]. Accordingly, the ultimate goal of corrective surgery for ASD is to restore adequate LL for sagittal balance and achieve solid arthrodesis. Although some postoperative sagittal spinopelvic parameters were significantly improved compared with the preoperative values in both groups, the P-A group showed a significantly greater increase in the correction amount of LL and C7SVA than the A-P group at the final follow-up (*p* < 0.05). Furthermore, the changes in SL at L2-3, L3-4, and L4-5 were significantly greater in the P-A group than in the A-P group (*p* < 0.05). Notably, the mean difference in correction amount of LL was approximately 9.3° between the two groups. Except for L5-S1, the sum of the mean difference in improvements of SL was approximately equal to 8° at L1–5 levels between the two groups. This finding is believed to be associated with increased spinal flexibility due to posterior osteotomies before ACR.

The PI is an individual and position-independent anatomical spinopelvic parameter, and the values of PI and LL have been demonstrated to show a strong positive association [58]. In clinical practice, PI − LL matching is a vital tool for obtaining optimal sagittal alignment during deformity surgery [4]. While the postoperative PI − LL mismatch significantly improved in both groups, the P-A group had a better value (2.9 vs. 13.4°, *p* < 0.001) and an improved curative effect than the A-P group. In the A-P group, 20 patients (24%) manifested postoperative PI − LL mismatch ≥ 10°, indicating that the deformity was not sufficiently corrected. This finding was surprising because previous studies [18, 59] demonstrated that the traditional A-P procedure provided restorative capacity similar to other techniques. Certainly, this finding does not demonstrate that the P-A procedure is superior to the A-P procedure in patients with ASD. Among the patients who underwent the A-P procedure, a significant correction was noted in the remaining 62 patients (76%) during follow-up, who were all identified with postoperative PI − LL mismatch < 10°.

To date, few reports have investigated the sequence of combined AP surgery. Turner et al. [60] reported that posterior osteotomies at ACR levels provided greater correction, but the sequence of surgical procedures was not mentioned. These findings indicate the importance of posterior spinal osteotomy in the treatment of severe sagittal imbalance. Particularly in patients with multilevel lumbar foraminal stenosis, posterior decompression combined with partial removal of the tip of the SAP may reduce the incidence of postoperative neurological deterioration secondary to foraminal stenosis after ACR in traditional combined A-P surgery. In the current study, two patients in the A-P group experienced neurological deterioration after the first stage surgery, and postoperative magnetic resonance image (MRI) revealed obvious compression of the nerve root in the neural foramina, although no significant compression was observed preoperatively. Thus, careful consideration of a patient’s underlying characteristics before surgery may prove valuable in surgical decision making.

Osteoporosis, which is a common disorder of the skeletal system characterized by decreased mechanical endurance of the bone and increased risk of fractures, is another important factor to be considered. This finding is consistent with the conclusions of previous studies showing that poor bone quality is associated with an increased incidence of cage subsidence. Unsurprisingly, decreased BMD leads to a lower failure load of the vertebrae, leading to a greater risk of cage subsidence, especially in patients with severe osteoporosis. In this study, the author found that posterior spinal osteotomy prior to ACR could improve the compressive strength and decrease cage subsidence at the index level, although osteoporosis is common in patients with ASD. In addition, the negative correlation between cage subsidence and insufficient LL correction is well-recognized.

The present study had several limitations, mainly because of its retrospective nature, small sample size, and short follow-up period, particularly when comparing the A-P group. Further studies with larger sample sizes are necessary to verify our results. In addition, this study was limited to radiographic outcomes and lacked PRO. Thus, a correlation between spinopelvic radiographic parameters and clinical outcomes to assess the impact of sagittal alignment on pain-related disabilities could not be established. Based on these findings, we believe that the results from this study may be utilized to permit refinement in the surgical planning for ASD and help surgeons better anticipate risks and threats, thus improving patient satisfaction.

### Conclusion

Posterior spinal osteotomy prior to ACR offers greater LL correction than the traditional combined anterior-posterior procedure. Indications of this approach procedure for ASD include patients aged ≥ 75 years, severe osteoporosis, rigid lumbar curve with dynamic changes in LL < 10°, or more than four facet joints of Pathria grade 3 in the lumbar region.

## Data Availability

The datasets used and/or analyzed during the current study are available from the corresponding author on reasonable request.

## Abbreviations

ASD: Adult spinal deformity
ACR: Anterior column realignment
LLIF: Lateral lumbar interbody fusion
ALL: Anterior longitudinal ligament
SL: Segmental angle
LL: Lumbar lordosis
C7SVA: C7 sagittal vertical axis
PT: Pelvic tilt
PI: Pelvic incidence
PI − LL mismatch: PI minus LL mismatch
PACS: Picture archiving communication system
BMI: Body mass index
CCI: Charlson comorbidity index
ASA: American society of anesthesiologists
BMD: Bone mineral density
UIV: upper instrumented vertebra
EBL: Estimated blood loss
PJK: Proximal junctional kyphosis
PJA: Proximal junctional angle
CT: Computed tomography
TK: Thoracic kyphosis
TLK: Thoracolumbar kyphosis
ICC: Intraclass correlation coefficient
OA: Osteoarthritis
SAP: Superior articular process
PRO: Patient-reported outcomes
OR: Odds ratio
CI: Confidence interval
PEEK: Polyetheretherketone
ROM: Range of motion
MRI: Magnetic resonance image
